# Charge Transport inside TiO_2_ Memristors Prepared via FEBID

**DOI:** 10.3390/nano12234145

**Published:** 2022-11-23

**Authors:** Markus Baranowski, Roland Sachser, Bratislav P. Marinković, Stefan Dj. Ivanović, Michael Huth

**Affiliations:** 1 Physikalisches Institut, Goethe University, 60438 Frankfurt am Main, Germany; 2 Institute of Physics Belgrade, University of Belgrade, Pregrevica 118, 11080 Belgrade, Serbia

**Keywords:** memristor, FEBID, titanium dioxide, resistive switching, SEM image, current-voltage (*i-v*) curves, temperature dependence measurements, Mott-type variable-range hopping

## Abstract

We fabricated memristive devices using focused electron beam-induced deposition (FEBID) as a direct-writing technique employing a Pt/TiO_2_/Pt sandwich layer device configuration. Pinching in the measured current-voltage characteristics (*i-v*), the characteristic fingerprint of memristive behavior was clearly observed. The temperature dependence was measured for both high and low resistive states in the range from 290 K down to about 2 K, showing a stretched exponential behavior characteristic of Mott-type variable-range hopping. From this observation, a valence change mechanism of the charge transport inside the TiO_2_ layer can be deduced.

## 1. Introduction

First postulated by Leon Chua [1] and practically realized in HP Labs [2], the memristor was originally considered the fourth basic two-terminal circuit element defined by the relationship between the instantaneous value of the electric charge and the instantaneous value of the flux linkage, i.e., a nonlinear relationship between the integrals of the current and voltage. This assertion has later been refuted [3]. In addition to the nonlinear element of memristor, the concept has been generalized to a broader class of nonlinear dynamical systems [4]. The Strukov–Williams model [2] has been widely used to describe the existence of high (HRS) and low (LRS) resistivity states of memristor devices based on a TiO_2_ layer sandwiched between two electrodes. Depending on the polarity of applied voltage, the barrier between an insulating sub-layer of TiO_2_ and an oxygen-deficient sub-layer TiO_2−x_ (oxygen vacancies are introduced by an irreversible forming process) is shifted, causing a switching event to occur at the rectifying non-ohmic interface. In simple terms, a memristor is a device in which resistance depends on the value, polarity, and duration of an applied voltage across its terminals; it also keeps a memory of the last existing resistance. It is characterized by the memristance, an observable that depends upon the internal state of the device, i.e., a state variable. The fingerprints of memristors have been introduced in the early paper on memristive systems [5] in order to define constitutive relations of the circuit element. One of the main characteristics of a memristor is its pinched (zero-crossing) loop hysteresis curve in the *i-v* plane under sinusoidal excitation and the behavior of the hysteresis curve upon frequency and input amplitude changes [6].

The mechanism of electroforming metal oxide memristive switches [7] is explained as an electro-reduction process in a large electric field that results in the creation of oxygen vacancies which, as they drift, are forming localized conducting channels. Electrical current passing through oxide (nano) material between conductive electrodes is then the result of a coupled electron and ion motion (drift) and tunneling in the oxide layer. Resistive switching phenomena have been recognized long ago and substantial efforts have been put into revealing the microscopic phenomena that govern the change from an insulator to a conductor. A comprehensive review of resistive switching in oxides, the role of oxygen vacancies, and the creation and rapture of conducting channels have been presented in [8]. Another recent review covers the whole area of the newly developed field of biomaterial-based nonvolatile resistive memory devices [9] in which the resistivity switching mechanism is considered one of the important examples where ionic motion creates an underlining mechanism of operation. This mechanism has been demonstrated to be useful in new optoelectronic devices that can be controlled both optically and electronically [10]. It has been already a decade of 2D-materials-based resistive random-access memories, and their progress is covered in a comprehensive review [11].

The intrinsic properties of memristor devices make them suitable for many different applications ranging from non-volatile resistive switching memories [12], applications in RF/microwave circuits [13], novel non-von Neumann computer architectures with memristive memory arrays performing in-memory computation [14,15], modeling of nonlinear systems with cellular neural networks and memristors as locally-active nonlinear elements [16], and the use of memristive devices for neuromorphic computing [17,18]. Although titanium dioxide has desirable properties for memory elements, such as retention and endurance, it seems that its real potential in applications lies in advanced areas of mimicking the biological synapses as a tool for creating artificial intelligence [19].

The first practical realization of a memristor was performed with TiO_2_ [2] by using electron-beam evaporation for metal deposition and sputter deposition or atomic layer deposition to make the titanium dioxide film, while an annealing process was performed in order to create oxygen vacancies in part of the TiO_2_ film. Not long after, physical characterization of Pt/TiO_2_/Pt unipolar [20] and bipolar [21] resistance-switching devices revealed, in detail, the mechanism of the formation of a Ti_4_O_7_ Magnéli phase which possesses metallic properties and ordered planes of oxygen vacancies [21], and confirmed that switching occurs by the formation and disruption of Magnéli phase filaments [20]. Another realization of an electroforming-free titanium dioxide memristor was performed as a metal-insulator-metal (MIM) crossbar device fabricated on a silicon/silicon-nitride substrate with the bottom and top electrodes consisting of Cr(5 nm)/Pt(15 nm) and Pt(30 nm), respectively, patterned in a cross-type junction form by photolithography [22]. Even an ink-jet printed fabrication technique for TiO_2_-based memristors has been demonstrated recently [23,24]. In [23], a vertical sandwich architecture of ITO/TiO_2_/Ag was exploited where the active layer was deposited in the form of nanoparticle ink over an indium tin oxide (ITO) coated glass substrate. A top thin layer of silver was used as an active anode while the ITO layer, when the voltage was applied and the heat was generated, affected the formation and destruction of the conductive filaments within the switching device. Another fully printed and flexible memristor has been demonstrated recently [25], as fabricated by depositing a thin film of metal-non-metal doped TiO_2_ that exhibited enhanced performance with self-rectifying and formed free bipolar switching behavior.

In this work, we present a TiO_2_-based memristor device fabricated by a direct-write approach using FEBID (Focused Electron Beam Induced Deposition) for both the Pt electrodes and the TiO_2_ switching layer. We have performed electroforming processes by in situ application of a controlled train of current pulses through the nanodevice and a shunt resistor and recorded the switching phenomena and hysteresis curves in the *i-v* plane. Resistance changes by a factor of up to 4.79 between the HRS and LRS states have been observed. We analyzed the measured temperature dependence of the *i-v* curve in the range from room temperature down to about 2 K for both the HRS and LRS states of the device, in order to reveal the conducting process, which we identified as activated tunneling. The main advantage of this novel approach is its applicability to virtually any surface on which memristive functionality is desired, as well as the down-scaling capability of the FEBID process, which allows for the realization of nano-scale memristive devices.

## 2. Materials and Methods

**Figure 1:**
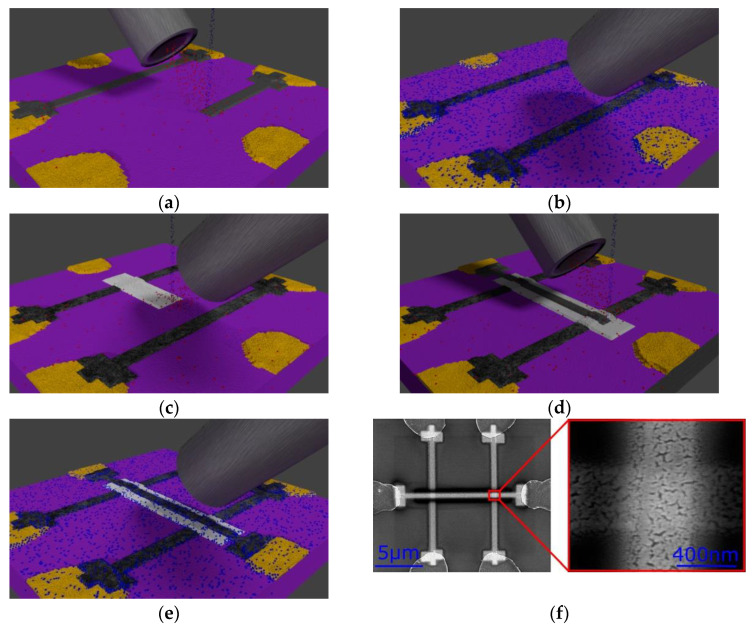
Graphical representation of the preparation process via FEBID: (**a**) Deposition of the Pt-C bottom electrodes; (**b**) Purification of the Pt-C bottom electrodes; (**c**) Deposition of TiO_2_ via FEBID; (**d**) Deposition of Pt-C top electrodes; (**e**) Purification of the Pt-C top electrodes; (**f**) SEM image of the double-TiO_2_ memristor device. On the left side: The whole structure which was deposited via FEBID is shown. One of the two sections where the memristor is located is highlighted with a red rectangle. On the right-hand side: The memristor (Pt/TiO_2_/Pt stack) is shown with higher magnification and reveals the nano-porous microstructure of the purified Pt-C electrodes.

The preparation of the TiO_2_ memristors is schematically illustrated in Figure 1a–e. To prepare the memristors, first, Pt-C bottom electrodes were deposited via FEBID. This is illustrated in Figure 1a and described in detail in Section 2.1. Afterward, the Pt-C bottom electrodes were purified to increase the Pt content of the deposits and, therefore, improve their electrical conductivity. This is explained in more detail in Section 2.2 and it is shown schematically in Figure 1b. Next, a thin layer of TiO_2_ was prepared on top of the Pt bottom electrodes, as indicated in Figure 1c. This process is described in Section 2.3. After that, a top electrode of Pt-C was deposited on top of the Pt/TiO_2_ structure, which is shown in Figure 1d. Afterward, the Pt-C top electrodes were again purified, which is shown in Figure 1e. An SEM image of two such fabricated memristor devices is shown in Figure 1f.

For a detailed account of FEBID, we refer to recent reviews, such as, for example, [26,27]. As a consequence, only a brief description of its utility needs to be given in the following subsections, with emphasis on those experimental procedures which are specific to the device fabrication. To measure the TiO_2_ memristors electrically, UV-lithography was used to prepare Cr/Au contacts on top of a Si (100) + 200 nm thermal SiO_2_ substrate. Afterward, Pt electrodes were deposited between the predefined Cr/Au contacts. The scanning electron microscope (SEM) which was used is an FEI NOVA NanoLab 600, equipped with a Schottky-type emitter operating at a base pressure of 5 × 10^−7^ mbar.

### 2.1. Pt-C FEBID Deposition

Trimethyl(methylcyclopentadienyl)platinum(IV) was used as a precursor for Pt-C electrode deposition. For the deposition of the Pt-C electrodes, the precursor was heated to 44 °C. Then, the supply to the substrate surface was achieved via a capillary that has an inner diameter of 0.5 mm at an angle of 52° to the sample surface. The capillary was placed 100 μm above the surface and 150 μm off-center of the focal point. The acceleration voltage of the electron was 5.0 kV at a beam current of 1.6 nA. A pitch of 20 nm, a dwell time of 1 μs, and 1566 passes were used. The pressure of the SEM chamber during the deposition was 1 × 10^−5^ mbar.

The size of the resulting electrodes was 15 μm × 0.5 μm × 10.6 nm for the bottom electrodes. The top electrodes had a size of 14 μm × 0.5 μm × 10.6 nm. The specified thickness of the PtC deposit is the thickness after the purification process.

### 2.2. Purification Process

After deposition the Pt-C FEBID electrodes were purified by heating the sample to 150 °C and exposing it to a pulsed O_2_ flow for 5 min, which was repeated twenty times, each time followed by a 5 min pumping period; see [28] for details.

The O_2_ for the purification process was delivered by a self-constructed gas injection system (GIS). The inner diameter of the homemade GIS is 0.5 mm at an angle of 15° to the sample surface. It was placed 100 μm above the surface and 150 μm off-center.

### 2.3. TiO_2_ FEBID Deposition

To prepare the TiO_2_ on top of the Pt bottom electrodes, titanium isopropoxide (Ti{OCH(CH_3_)_2_}_4_) was used as a precursor, employing the same self-constructed gas injection system. To deposit the TiO_2_ an acceleration voltage of 5.0 kV, a beam current of 1.6 nA was used. During the deposition, a pitch of 20 nm, a dwell time of 1 μs, and 3500 passes were used at a partial pressure inside the SEM chamber of 1.3 × 10^−5^ mbar. The thickness of the TiO_2_ deposit was about 10 nm. To estimate the thickness of the TiO_2_ layer, an energy-dispersive X-ray analysis (EDX) was used. Since EDX is not designed to measure thickness but to measure the composition of a material, the calculated thickness of the TiO_2_ is an indirectly obtained measure of the actual thickness. The basic approach here is that if a measured material is thin enough to see characteristic X-rays from the substrate material in the EDX measurement, meaning in our case SiO_2_, then an EDX measurement can be used to calculate the thickness as follows. This was performed by measuring the percentage of Si via EDX for different thicknesses of TiO_2_ deposits and also measuring the thickness of these deposits independently via AFM. With both these parameters (thickness of the deposits measured via AFM on one hand and the percentage of Si, which is obtained via EDX composition analysis), an estimated thickness was calculated. The thickness has been measured by AFM in order to make a calibration curve and to know how many passes of the electron beam correspond to which thickness in the deposition. The process is much more effective for Pt deposition, which depends on the available precursor molecular target.

### 2.4. Forming the Memristor

**Figure 2:**
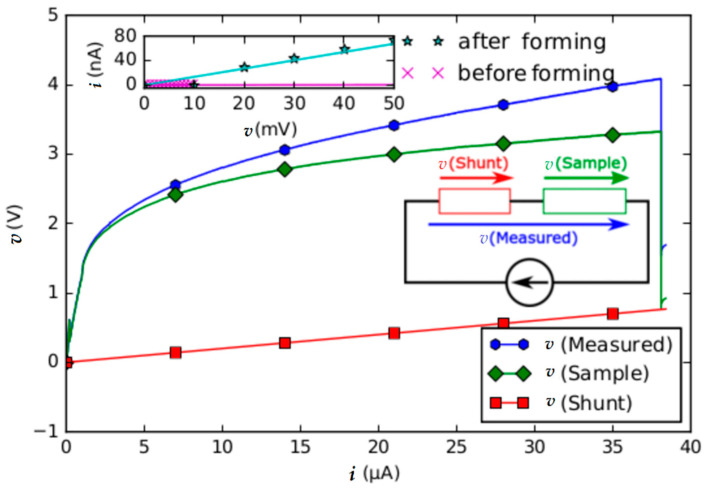
*i-v* curve of a TiO_2_ memristor during the forming process. *v*(Measured) contains the resistance of a Pt/TiO_2_/Pt stack plus a shunt of 20 kΩ which is connected in series. From this, the actual voltage drops at the memristor, and the shunt can be calculated which are indicated as *v* (Sample) and *v* (Shunt), respectively. The sudden voltage drop at approximately 3.7 V corresponds to a partial dielectric breakdown of the TiO_2_ and forms the memristor out of the Pt/TiO_2_/Pt stack. The inset shows extrapolated *i-v* data of the memristor before and after forming.

In order to create a memristor from the stacked layers of Pt/TiO_2_/Pt, oxygen vacancies have to be introduced into the TiO_2_. These vacancies can be created by applying an electric field higher than the breakdown voltage of TiO_2_. A typical *i-v* characteristic of the forming process is shown in Figure 2. To form a memristor, we found that an electric field of 340 V/μm was needed. This is higher than the breakdown voltage of TiO_2_ previously deposited via FEBID, which was found to be between 10 V/μm to 70 V/μm [29]. However, the breakdown voltage value can vary significantly with various parameters, including thickness, deposition method, purity, and the area of the TiO_2_ layer. For our sample geometry, which is shown in Figure 1f, the applied electric field led to the current densities of 720 × 10^3^ A/cm^2^ for the Pt electrodes and 15 × 10^3^ A/cm^2^ for the TiO_2_. Such current densities can only be tolerated by the cross-type devices if the forming process is performed via a pulsed measurement.

The current duty cycle in the pulsed measurement was 10 μs, during which the associated voltage was recorded. In between the current pulses, a waiting time of 100 μs at zero current was introduced. This was repeated for each current value 10 times for a better signal-to-noise ratio.

### 2.5. Transport Measurements

The electric measurements were performed with a Keithley source-meter 2400. To measure the electric conductivity, a standard two-probe measurement with a shunt in series with the sample was used. The shunt was necessary to limit the current density in the samples. As described, the electric conductance measurements were performed via pulsed measurements in order to limit the dissipated power in the samples. The temperature-dependent electrical conductivity measurements were performed in the temperature range between 2 K and 300 K inside a ^4^He cryostat using a variable temperature insert. The cooling rate was set to 1.5 K/min. The conductance has been measured as the ratio of current to voltage for each data point. During low-temperature measurements, a fixed voltage of 10 mV was used and the output current was recorded to determine the conductance. The number of power line cycles (NPLC) was usually set at 0.1, but some measurements were performed with 0.01 and 1.

## 3. Results

**Figure 3:**
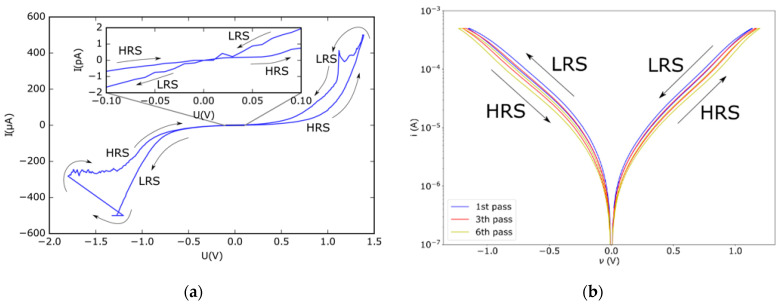
The *i-v* curve of the formed Pt/TiO_2−x_/Pt device. The pinching, a characteristic of memristors, is apparent in the measured *i-v* data. (**a**) The first stable *i-v* curve after forming was measured on sample 821. The inset shows the *i-v* data near zero bias in detail; (**b**) The *i-v* curve after several cycles was measured on sample 822.

The formation of a memristor from the Pt/TiO_2_/Pt stack was found to reduce the resistance from 116 MΩ before the forming process to a resistance of 613 kΩ after the forming process. This corresponds to a decrease in the resistance by a factor of 190. The *i-v* curves before and after forming of the memristor are shown in the inset of Figure 2. After the forming step, the memristive behavior of the Pt/TiO_2−x_/Pt stack has to be demonstrated. This can be performed by reference to the shape of the *i-v* curve. Following Chua [30], a memristor is characterized as a 2-terminal device that shows a "pinched hysteresis loop confined to the first and third quadrants of the *i-v* plane whose contour shape in general changes with both the amplitude and frequency of any periodic sine wave-like input voltage source, or current source”. For our Pt/TiO_2−x_/Pt stack, the *i-v* dependence was measured and is shown in Figure 3 for two samples, where the formation of a pinched hysteresis loop is apparent. The inset shows the difference between the high resistive state (HRS) and the low resistive state (LRS) near zero bias in detail. The difference between the LRS and the HRS corresponds to a ratio of 4.79 at 70 mV. Considering the range around zero bias of the *i-v* curve in more detail, after several cycles of changing from the HRS to the LRS, the occurrence of several states instead of only two becomes apparent. This is another characteristic of memristor devices [30]. Figure 3 shows a characteristic behavior of reversible bi-polar nonvolatile switching loops where increasing voltage induces set switching from HRS to LRS and reset switching from LRS to HRS. These two states are represented by different slopes of *i-v* curves.

**Figure 4:**
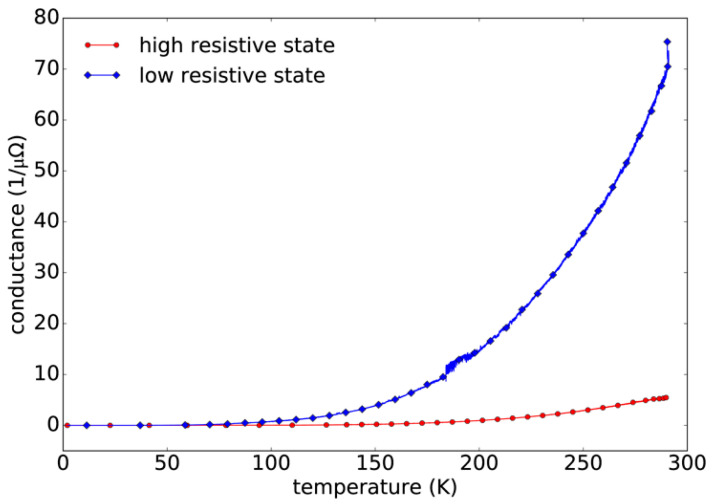
Measured temperature dependence of the conductance of the LRS and the HRS. Both states show a thermally activated behavior.

To identify the charge transport mechanism inside the TiO_2_ layer for the LRS and the HRS, we monitored the temperature dependence of the conductivity in both states. This is shown in Figure 4, where a thermally activated behavior of the LRS and the HRS inside the TiO_2_ is visible.

## 4. Discussion

There are several resistive switching mechanisms possible that may explain the observed conductance of memristive devices depending on the materials and interfaces. The resistance regime can be interface-controlled due to Schottky emission, or the dominant conduction can be changed from an interface to a core-material-controlled mechanism [31]. For TiO_2_, there are two known dominant mechanisms, namely the valence change mechanism and the thermochemical mechanism [32]. Both mechanisms lead to the formation of a conductive path through the insulating TiO_2_, thereby decreasing the resistance locally. In the case of the valance change mechanism, the conducting path consists of O_2_ vacancies. In the case of the thermochemical mechanism, the conductive path consists of a Ti_4_O_7_ Magnéli phase [20,21].

**Figure 5:**
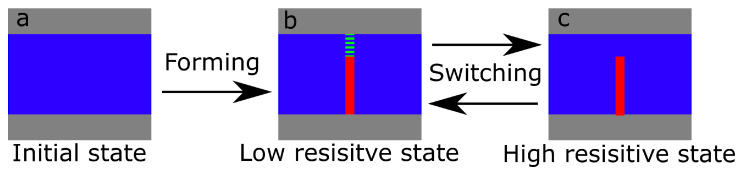
Schematic representation of the forming process in the Pt/TiO_2_/Pt stack: (**a**) The initial state where the top and bottom layers represent Pt electrodes and the middle layer represents TiO_2_; (**b**) The situation after forming where oxygen vacancies form conducting paths through the whole sample. This state is called the low resistive state (LRS); (**c**) The LRS state can be switched to the high resistive state (HRS) where the conducting path created by oxygen vacancies does not connect the top and bottom electrodes.

Experimentally, it is possible to determine which kind of mechanism is at work in a TiO_2−x_ memristor, because the valance change system shows bipolar switching in the *i-v* curve after the forming step, while the thermochemical systems exhibit unipolar switching [32]. In our case, the memristors exhibited exclusively bipolar switching in the *i-v* curve, as shown exemplarily in Figure 3. We, therefore, conclude that the underlying mechanism for the forming and subsequent switching of the TiO_2−x_ memristors is due to the valence change mechanism, as is schematically illustrated in Figure 5.

Application of a positive voltage to the bottom electrode induces oxygen vacancy migration toward the top electrode, resulting in conductive filament formation and growth. This is a filamentary switching in metal oxides (in our case TiO_2_), which is based on features of conductive filaments, where localized channels with a lower concentration of oxygen have a higher electrical conductivity [33,34]. A set transition occurs at a positive voltage, while a reset transition is operated at a negative voltage. To avoid a destructive breakdown during the set process, the current has to be limited.

**Figure 6:**
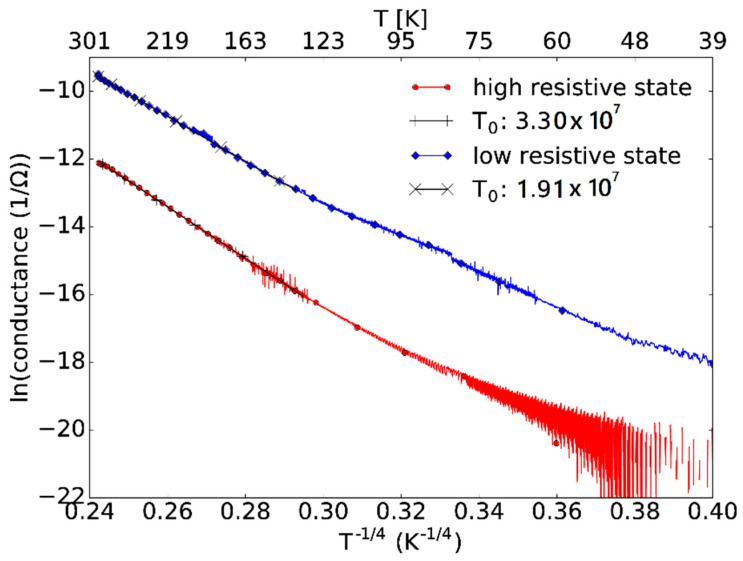
Temperature-dependent conductivity of a fabricated memristor placed in the cryostat and measured for the HRS (red) and LRS (blue) states.

This assumed mechanism is further supported by the temperature-dependent conductance measurements which are shown in Figure 6. When the temperature-dependent conductance data are plotted assuming a Mott-type variable-range hopping mechanism, a *T*^−1/4^ dependence of the conductance in logarithmic representation between 135 K and 290 K is observed. This further supports the assumption that it is a valence change system where the reason for the electrical transport is due to oxygen vacancies. The temperature dependence shows a Mott-type variable range hopping where the conductivity is varying as $\sigma =\sigma_{o}e^{-{\left(T_{o}/T\right)}^{1/4}}$. The parameter $T_{o}$ for the high and low resistive states was found to be 3.30 × 10^7^ K and 1.91 × 10^7^ K, respectively.

**Figure 7:**
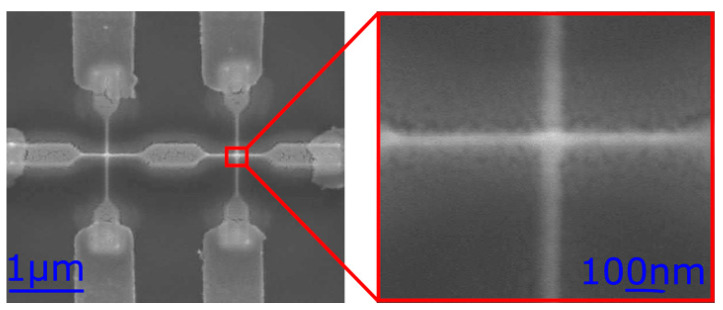
On the left-hand side is an SEM image of two smaller memristors with their accompanying Pt and gold contacts. The red rectangle is placed over one memristor, which is shown on the right-hand side in detail. The bottom and top Pt electrodes are seen clearly. Between these two electrodes is the TiO_2_. The widths of the Pt electrodes are approximately 48 nm.

In order to demonstrate the down-scaling capabilities of our direct-write approach, smaller structures were prepared which are shown in Figure 7. However, further optimization of the deposition and the purification procedure is necessary for reliable memristive behavior necessary for such devices with cross-section dimensions below 50 nm. Consequently, all electrical characteristics shown here refer to the larger memristor devices, as shown in Figure 1f.

The comparison of the fabricated memristor device with other memristors needs to take into account that our device has not been optimized for a specific use, which would demand further technological studies. Additionally, there is a wide range of applications where memristors are utilized. Their implementation is suitable due to their small dimensions, low power consumption, and fast switching times. Different models have been employed to cover a wide range of applications [35]. The range of operating values of resistance of our memristor is from 3 kΩ to 600 kΩ and capacitance is from 0.1 fF to 10 fF, depending on the thickness and purity of the TiO_2_ layer and the total area of the device forming a capacitor. We can compare these values with some of the values of Pt/TiO_2_/Ru memristors produced by the atomic-layer deposition process in [36], where resistance varies from 30 Ω to 600 kΩ and estimated capacitance of 1000 fF. Pi et al. [37] fabricated a memristor for RF/microwave applications at frequencies from 10 MHz to 110 GHz consisting of a Ti adhesion layer on an intrinsic silicon wafer with electrodes separated by a 35 nm-wide air gap. Their average values used for a model simulation are 3.6 Ω for a low resistivity state and a capacitance of 1.37 fF (estimated basically from the capacitance of the air gap, which depends on the effective dielectric constant of the substrate–air interface) for a high resistivity state.

## 5. Conclusions

In conclusion, a novel way to prepare memristors (via FEBID) has been shown. Using FEBID to prepare the memristor offers a highly flexible approach and can be used to place the memristors on top of already existing structures.

The memristors were formed by applying a voltage greater than the breakdown voltage using the pulse-delay technique. This led to two states, a high resistive state (HRS) and a low resistive state (LRS). Both states were characterized via temperature-dependent transport measurements which showed that the conduction of the memristors can be described by a Mott-type variable-range hopping in a temperature range between 135 K and 290 K.

Our standout features are (i) a rapid prototyping approach, (ii) the applicability to almost any surface, i.e., the option to add a memristive device to other pre-existing structures, (iii) the option for direct in situ device characterization, and, based on the results, optimization of the fabrication process for better device performance. Additionally, we have demonstrated only one possible FEBID-based approach with Pt/TIO_2_/Pt. Other material combinations are certainly feasible by tapping the base of growing materials available by FEBID. Mass production of memristive devices, on the other hand, is hard to envision based on FEBID, although semi-automatic device fabrication on a medium scale is feasible. This study complements the large body of literature on memristive devices and the different methods of their manufacturing, following our understanding of transport mechanisms and providing numerous applications [38].

## Data Availability

The data presented in this study are available on request from the corresponding author.
